# Practical Implications in Contemporary Dental Aesthetics—Shade Selection Assessment Using Intraoral Scanners

**DOI:** 10.3390/dj13010043

**Published:** 2025-01-20

**Authors:** Alice Arina Ciocan Pendefunda, Cristina Gena Dascalu, Sebastian Bahrim, Cristina Iordache, Odette Luca, Magda-Ecaterina Antohe

**Affiliations:** 1 Department of Odontology—Periodontology, Fixed Prosthodontics, Faculty of Dental Medicine, “Grigore T. Popa” University of Medicine and Pharmacy, 16 Universității Street, 700115 Iasi, Romania; alice.ciocan@umfiasi.ro; 2 Department of Medical Informatics and Biostatistics, Faculty of Medicine, “Grigore T. Popa” University of Medicine and Pharmacy, 16 Universității Street, 700115 Iasi, Romania; 3 Department of Implantology, Removable Restorations and Technology, Faculty of Dental Medicine, “Grigore T. Popa” University of Medicine and Pharmacy, 16 Universității Street, 700115 Iasi, Romania; sebastian.bahrim@umfiasi.ro (S.B.); marina.iordache@umfiasi.ro (C.I.); elena-odette@umfiasi.ro (O.L.); magda.antohe@umfiasi.ro (M.-E.A.)

**Keywords:** aesthetics, intraoral scanner, tooth’s surface properties, dental shade

## Abstract

**Background/Objectives:** Aesthetics is a challenging aspect to restore for both dentists and dental technicians. One of the characteristics of aesthetic restoration is the shade. The purpose of the study is to assess the accuracy of the shade selection feature of intraoral scanners (CEREC Omnican, 3Shape TRIOS) in comparison with an already established method—the VITA Easyshade V spectrophotometer (VE)—and test if there is any significant difference between the three devices. **Methods:** To conduct this in vitro study, the VITA Classical shade guide was used. The intraoral scanners would not be able to scan the VITA Classical as it is, hence, a study model (SM) was fabricated. To be able to test the accuracy of the intraoral scanners (IOSs) in detecting the dental color, a spectrophotometer had to be included in the study, as it was shown that it is the most accurate instrument for this purpose. Therefore, for the current study, the VITA Easyshade V spectrophotometer (VITA Zahnfabrik, Bad Sackingen, Germany) was selected. **Results:** The accuracy of the three devices when measuring the shade of the study model was calculated as a percentage. When comparing the primary results of the VE and the results obtained by the Omnicam and TRIOS, the latter is the most accurate (26.67%), whereas the other two scored 20%. The study also revealed the limitations of the instrumental devices that were used. **Conclusions:** First, both the VE and IOSs obtained unexpectedly low accuracy results. Possibly, the material VC is made of influenced the final accuracy values, but in practice, on a daily basis, dental materials represent a factor that cannot really be controlled.

## 1. Introduction

Contemporary society is facing a spectacular evolution of the techniques and technologies involved in aesthetic rehabilitation, the correct quantification of the final color having a determining role in the final therapeutic success. The digital era is definitely influencing every level of restorative dentistry and is also reflected in the methods of dental shade identification.

Color is strongly related to light. In order to be able to see the colors of an object, the light reflected is perceived as a stimulus by the neural sensors of the retina. A signal will be further sent to the visual cortex in the brain that will interpret the information [1,2]. Incident white light’s reflected compounds determine the specific color of the object. For instance, transparent materials allow the light to pass through with minimum modifications. Light is scattered, transmitted, and absorbed by translucent materials. Opaque materials only reflect and absorb the light, without transmitting it. The natural tooth has a semi-translucent structure, which makes the shade selection process more difficult when comparing it to an opaque object. A tooth’s surface properties, such as gloss, angles, and texture, affect the way in which light diffuses when it strikes a specific object [3].

Aesthetics has been a challenging aspect to restore for both dentists and dental technicians. One of the characteristics of aesthetic restoration is the shade. First, there were visual shade guides whose purpose was to assist the dental professional in correctly choosing the tooth color. However, visual methods have been proven to be subjective and influenced by several factors [4,5].

The choice and reproduction of color in contemporary dental aesthetics can be associated with a true art based on scientific criteria that are found in both the clinical and technological register, being in direct interrelation with the brightness, the shape of the tooth, without eluding the composition of the chosen biomaterials [6].

“The curvatures of surfaces, the depth of spaces, the contours, all become visible as a result of the interplay of light and shadow. Light has this ability to change the perception of a surface as a result of its interaction with its shape”. Natural teeth reflect light relatively uniformly across their surface. Light reflection is influenced by the nature of the surface. A smooth surface will reflect light entirely, like a mirror, while a rough surface will induce a diffuse reflection of light on the one hand and cause partial absorption on the other hand [7].

The way light is reflected from tooth surfaces also influences the visual perception of teeth. On the curved surface of the buccal surface of the maxillary central incisor, only light rays falling perpendicular to it are reflected anteriorly. The rest of the rays will be reflected upwards or downwards depending on the orientation of the convexity of the surface. As a result, the human eye will only perceive the surface that reflects light anteriorly. This can create optical illusions suggesting that the tooth is elongated by enlarging this surface, or on the contrary, that the tooth is shortened by shortening the anteriorly reflecting surface [8].

The play of light and shadow creates contrasts and makes it possible to perceive shapes and colors. This phenomenon is evident in the case of the negative lateral space, the existence of which allows the contours of the lateral teeth to be perceived. These teeth will also give the impression that they are darker in color than the anterior teeth [9].

As an alternative, digital devices were introduced in dentistry. Spectrophotometry is considered, at the moment, the gold standard for dental color assessment [10]. Colorimeters, spectroradiometers, and computerized analysis of digital methods are other devices that studies showed can perform better than the traditional, visual methods [11].

Recently, intraoral scanners have also been used for this step in the restoration process. Their initial purpose was to replace the conventional impressions with faster, more accurate digital ones. However, the newer generations of IOSs from 3Shape and Dentsply Sirona are able to detect the dental shade using as reference the VITA shade guide. Until the current study, little research has been carried out on the accuracy of IOSs to determine the color of the teeth. So far, several conclusions have been presented: the 3Shape TRIOS did not perform as accurately as a colorimeter [12], and the same scanner had similar results as a spectrophotometer [11]. The latter conclusion was also reached in another study that also assessed the Cerec Omnicam and Primescan, but the two scanners from Dentsply Sirona showed a poorer accuracy compared to the spectrophotometer [13]. Therefore, the results can be considered inconclusive, and further studies, including the present one, are recommended. There are many studies that compared the visual and instrumental methods, and they showed, in most cases, better results for the dental spectrophotometer than the conventional, visual methods [14,15]. At the same time, there were studies that either obtained no significant difference between the two methods [16] or others that showed that visual color determination was more accurate in comparison to the instrumental method [17]. However, studies that assessed the property of intraoral scanners to determine shade are in limited number [18]. There were studies that showed that visual methods produced more accurate results compared to the VITA EasyshadeTM spectrophotometer [19]. In contrast, there was some research that indicated that instrumental methods (spectrophotometer, intraoral scanner) are more reliable than visual methods [20]. Regarding IOSs, a study compared an intraoral scanner (3Shape TRIOS) and a colorimeter. It concluded that the accuracy of the scanner to detect teeth color cannot be guaranteed; it did not perform significantly better than visual methods, and physicians should not solely rely on this method [21].

A successful aesthetic restoration does not mean only an appropriate shape and size, morphology, and translucency close to that of the natural tooth. Choosing the right color for the final restoration is also an essential aspect. It is a challenging process comprising three steps: shade selection, communicating the color to the laboratory, and reproduction of the natural tooth color. Dr. Steve Bergen affirmed that “Color is unimportant to the physiological success of dental restoration, yet it could be the controlling factor in overall acceptance by the patient”. Introduced in 1956, VITA Classical had been, for many years, a gold standard, and it still is a viable option for clinical shade assessment. Following Sproull’s recommendations regarding the ideal shade guide, VITA Zahnfabrik released, in 1998, the Toothguide Vitapan 3D-Master. The main difference between TG and VC is that the tabs in TG were systematically and logically organized, which was missing in VC. TG comprises 26 tabs distributed in five categories, according to value. The groups are numbered from 1 to 5, where the higher the number, the lesser the value. Within each group, the tabs have similar brightness. Chroma increases from up to down and it is numbered from 1 to 3. In comparison with the VITA Classical shade guide, the Vitapan 3D-Master offers the following improvements: a wider range of value, an increased number of tabs, the hue is orientated towards the reddish band of the spectrum, tabs are logically organized and more uniform, and the arrangement of the groups is better. Despite these benefits, the dentists or dental technicians that have less experience or do not possess enough knowledge about colors can find it difficult to understand the principles behind the Toothguide Vitapan 3D-Master. Moreover, given the fact that there are 26 tabs to choose from, it can increase the difficulty of the procedure for some dental professionals [21].

Data from the current literature provide information on the superiority of intraoral scans in identifying and matching dental shades, correlating with the chosen shade guides, with digital techniques being on a much higher level than visual shade matching. It is important to note that accuracy varied significantly between studies; one element of certainty is that setting the oral scanners to the Vita 3 The Master shade guide improved the accuracy of the recordings. Although a significant number of studies advocate the superiority of intraoral scans set to the Vita 3D The Master shade guide, visual verification of shade is also recommended and, therefore, further studies are needed to address the limitations of the current studies. From this perspective, we consider that our study addresses the use of state-of-the-art intraoral scans that guide the practitioner in the choices; we equally chose the classic key precisely for the high degree of usefulness in daily practice [22]. Therefore, there is no constant general conclusion yet regarding the ability of intraoral scanners to accurately detect the dental shade.

In this perspective, the current study aimed to assess the shade selection property of intraoral scanners in comparison to a dental spectrophotometer. The null hypothesis was that there is a significant difference between the accuracy of the spectrophotometer and the intraoral scanners in detecting the dental shade and no significant difference between the corresponding L*a*b values for each device determination.

The purpose of the study is to assess the accuracy of the shade selection feature of intraoral scanners (CEREC Omnican, 3Shape TRIOS) in comparison with an already established method—the VITA Easyshade V spectrophotometer (VE)—and test if there is any significant difference between the three devices.

## 2. Materials and Methods

**Figure 1:**
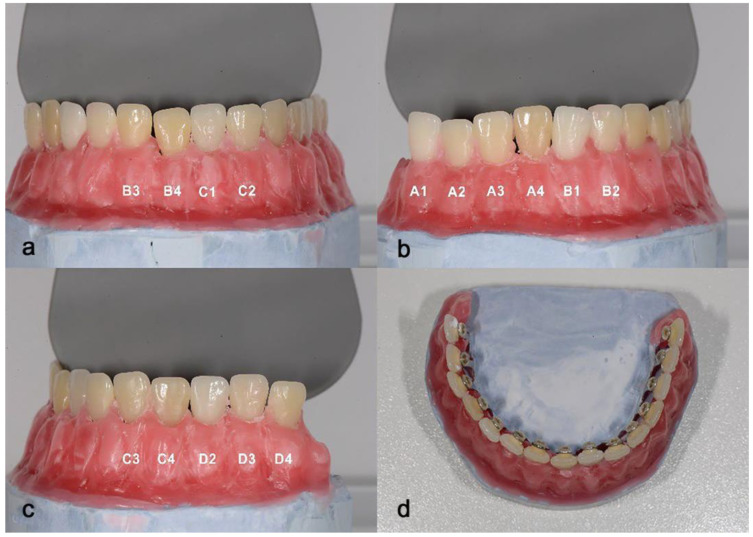
Study model. (**a**) Frontal view (tabs B3, B4, C1, C2). (**b**) Lateral view (A group, B1, B2). (**c**) Lateral view (D group, C3, C4). (**d**) Upper view.

To conduct this in vitro study, the VITA Classical shade guide was used. The IOS would not be able to scan the VC as it is, hence, a study model (SM) was fabricated. The VC tabs were removed from the guide. Class III blue plaster (Moldano, Kulzher, Hanau, Germany) and a model base mold were used to mount all 15 tabs. After four minutes of waiting, the plaster was soft enough to introduce the tabs but hard enough to stabilize them. Teeth were positioned in the same order as in the guide, in the shape of a dental arch. After 7 min, according to manufacturer instructions, the plaster set and was removed from the mold. Wax was added and modeled to recreate the aspect of the gingiva (Figure 1).

**Figure 2:**
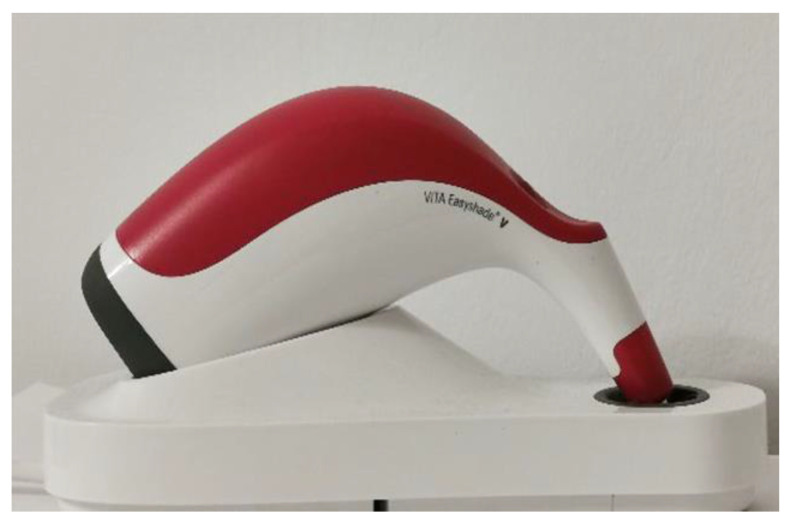
VITA Easyshade V.

To be able to test the accuracy of intraoral scanners in detecting the dental color, a spectrophotometer had to be included in the study, as it was shown that it is the most accurate instrument for this purpose. Therefore, for the current study, the VITA Easyshade V spectrophotometer (VITA Zahnfabrik, Bad Sackingen, Germany) was selected (Figure 2). The VITA is precise, fast, objective, simple, and intuitive to use. According to the manufacturer’s instructions, the VE is able to detect the color of both natural and artificial teeth. The VE was set to base shade mode. In this mode, the device is able to display the result according to the VITA Classical guide or L*a*b values. It displays the measured VC value, but it also suggests another value, the closest from the VITA Classical guide to the measured one. Therefore, the initial values were considered primary values, and the suggested ones were named secondary values. One measurement was taken for each tab, and both VITA Classical and L*a*b values were recorded. The tip of the VE was positioned in the middle of each tooth and ensured contact with the surface. Before each individual measurement, the device was calibrated by placing it in its dedicated support and waiting for the device to notify the end of this automatic process.

**Figure 3:**
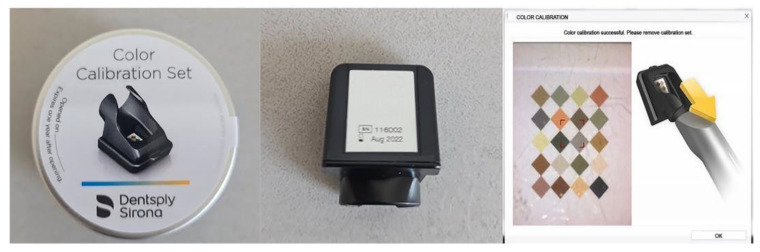
Cerec Omnicam color calibration set—required to successfully perform the calibration step before shade analysis.

**Figure 4:**
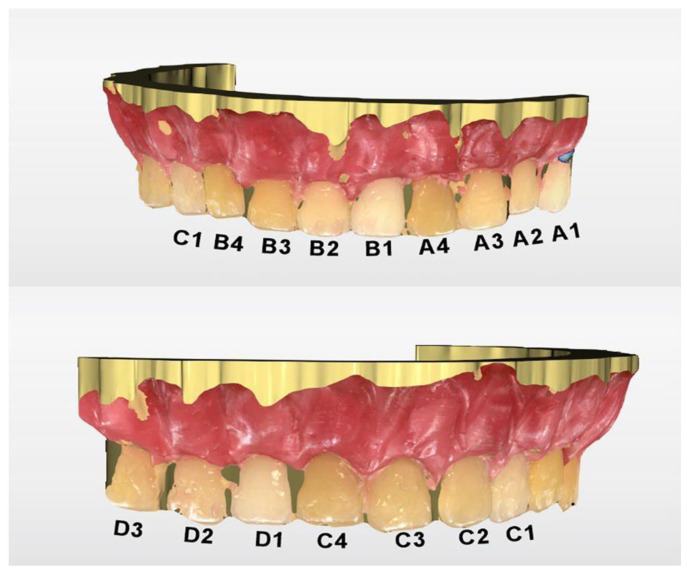
Digital model obtained with CEREC Omnicam SW 5.1—the initial shade tabs were marked.

The first IOS to be included was the CEREC Omnicam SW 5.1 (Dentsply Sirona, Bensheim, Germany). Since SW 4.5, this scanner has been able to detect the dental color. Therefore, it was used to scan the study model and analyze the shade. Color calibration was performed with the dedicated calibration tool (Figure 3) before proceeding with the scanning. After data were acquired, the software processed the scanning and delivered the digital model (Figure 4). Shade analysis was chosen from the available analyzing tools, and each tab group (A to D) was assessed. The shade radius was established to 2.0 mm, and the circle was placed in the middle third of each tooth. VITA Classical was chosen as the reference shade guide.

**Figure 5:**
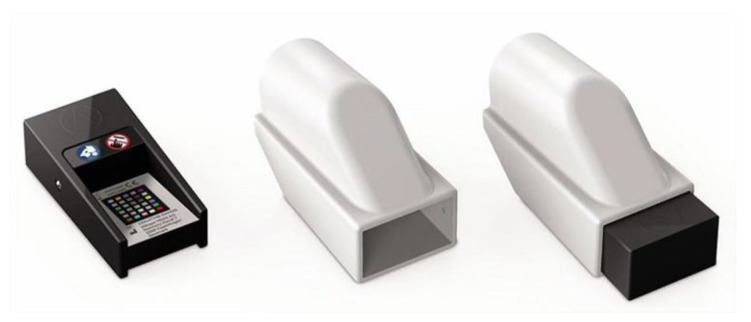
3Shape color calibration kit.

**Figure 6:**
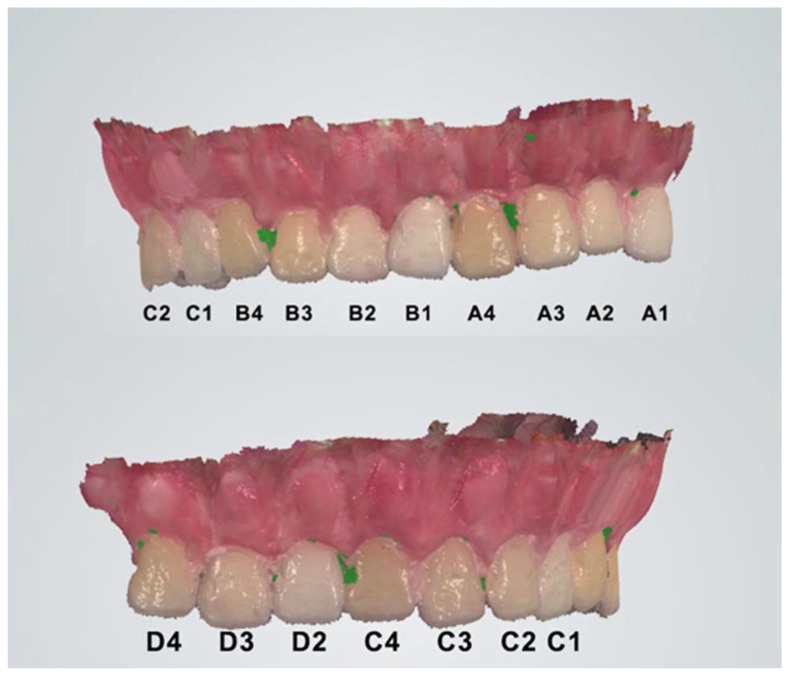
Digital model obtained with 3SHAPE TRIOS—the initial shade tabs were marked.

The second intraoral scanner that was selected for this study was the 3Shape TRIOS (3Shape, Copenhagen, Denmark). Since 2015, its software has been capable of detecting dental color. Similar to the other scanner, the TRIOS required color calibration before using this feature of the software. For this, the dedicated color calibration kit from the 3Shape was used (Figure 5). This kit did not have an expiration date as the similar kit for the Omnicam. After successful calibration, the study model was scanned with the scanner, and a digital model was obtained (Figure 6).

Using the software’s tool for color detection, the shades of the study model were determined from the middle third of each table. The 3Shape TRIOS refers at the same time to both the VITA Classical and VITA 3D-Master shade guides. However, for the purpose of the study, only VITA Classical analysis was taken into consideration.

**Figure 7:**
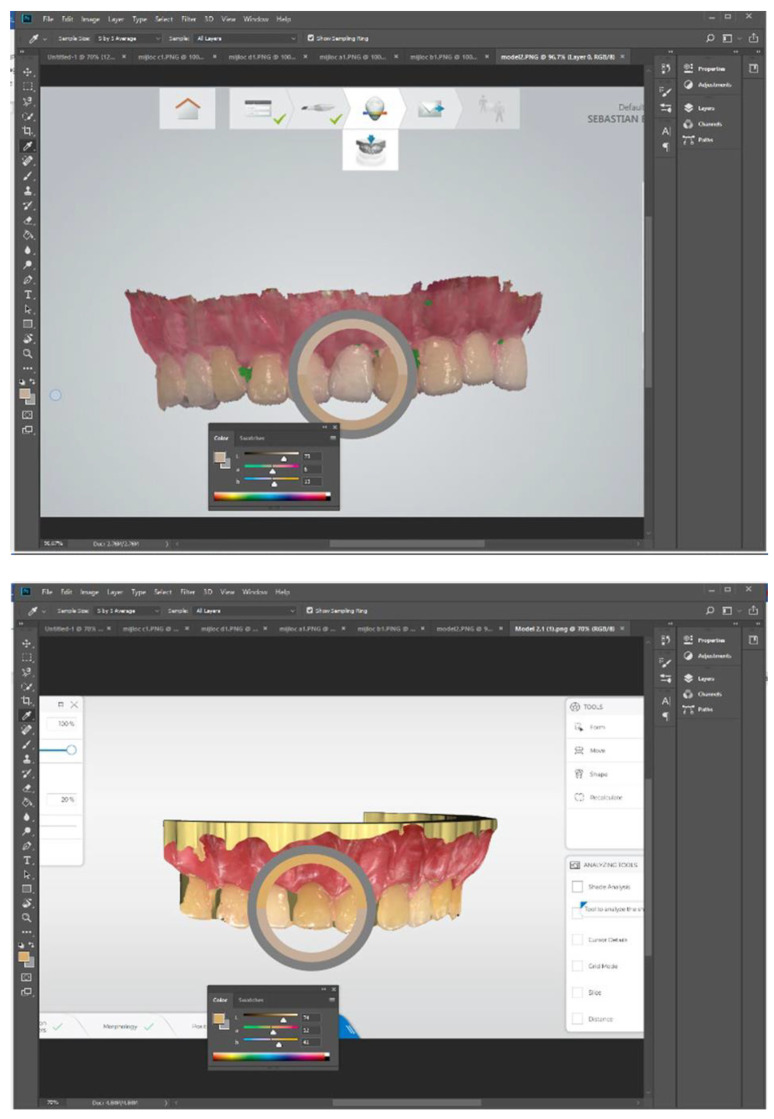
Adobe Photoshop CC 2018 to record CIELAB values from Omnicam (Dentsply Sirona, Bensheim, Germany) and TRIOS (3Shape, Copenhagen, Denmark) scan screenshots.

None of the intraoral scanners is able to deliver the CIELAB values for the shade tabs themselves to be directly compared to the L*a*b values generated by the spectrophotometer. Therefore, a screenshot was taken for each tab group from both digital color models and stored as a digital image file (portable network graphics—PNG). For the digital analysis of the pictures, Adobe Photoshop CC 2018 (Adobe Systems, San Jose, CA, USA) was opted for, which is one of the commercial software packages able to determine the CIELAB values from a picture. One by one, each screenshot was opened in the software. The eyedropper tool was used, a color sampler 5 by 5 average was set, and measurements were taken in the middle third of each tab (Figure 7).

**Figure 8:**
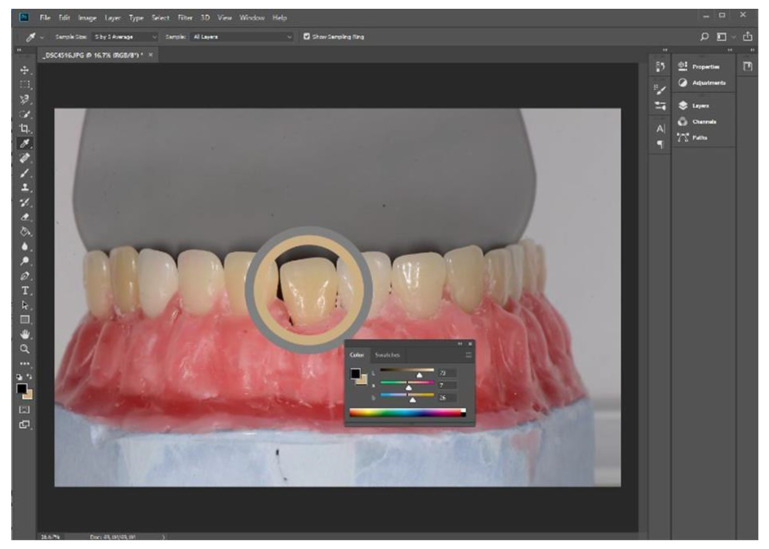
Adobe Photoshop CC 2018 to record CIE L*a*b values of shade tabs from digital pictures.

At the same time, the study model was photographed with a digital SLR with a 105 mm macro lens. An 18% grey card was placed behind the model as a contrast, and pictures were captured. The digital pictures were analyzed following the same steps as for the IOS screenshots (Figure 8).

All values were saved and processed in a Microsoft Excel spreadsheet. Four methods of obtaining results were established: VITA Easyshade (VE), Omnicam, TRIOS, and Photoshop.

The CIE L*a*b values recorded by the VE were compared with the values obtained through digital pictures analyzed using Adobe Photoshop. The color difference ΔE* for each shade tab recorded between the VE and each method was calculated using the following formula:

ΔE* = [(LV* − LX*)^2^ + (aV* − aX*)^2^ + (bV* − bX*)^2^]^1/2^

where LV*, aV*, and bV* are the measurements of the shade tabs obtained by the spectrophotometer, while LX*, aX*, bX* are the values obtained, one by one, by the Omnicam, TRIOS, and Photoshop.

Statistical analysis was performed in SPSS 29.0. Accuracy was determined as a percentage of the correct color match for each device for each shade tab, where VC values of the study model represent the reference point. The Pearson chi-squared test was used to check the differences between the percentages of matches for each method, and the one-way ANOVA test was used to determine whether there was any significant difference in measuring each of the L*a*b values of the VE in comparison to the other three devices. The single factor ANOVA tests were followed by post hoc Tukey’s HSD tests that assessed the relationship between each two of the four methods. Moreover, Pearson’s correlation test was used to check whether there is any correlation between L*a*b values provided by the VE in relation to the IOS and Photoshop values for each shade tab. The values of *p* < 0.05 were interpreted as statistically significant, while the values of *p* < 0.01 were interpreted as statistically highly significant.

## 3. Results

**Figure 9:**
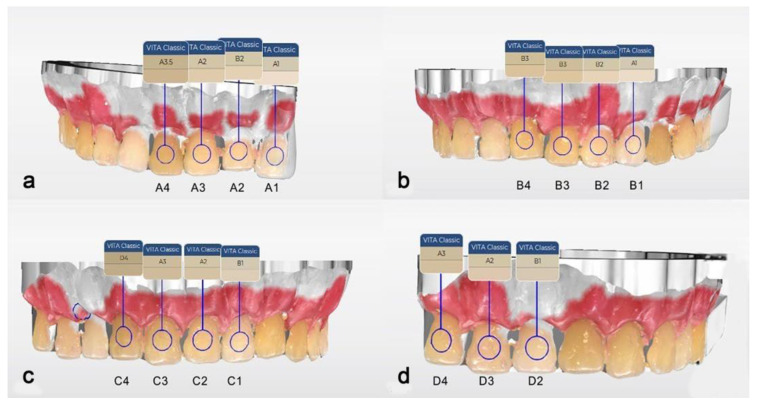
Shade selection of the middle third of the VC tabs using CEREC Omnicam IOS, according to VITA Classical Guide. (**a**) VC A group. (**b**) VC B group. (**c**) VC C group. (**d**) VC D group.

**Figure 10:**
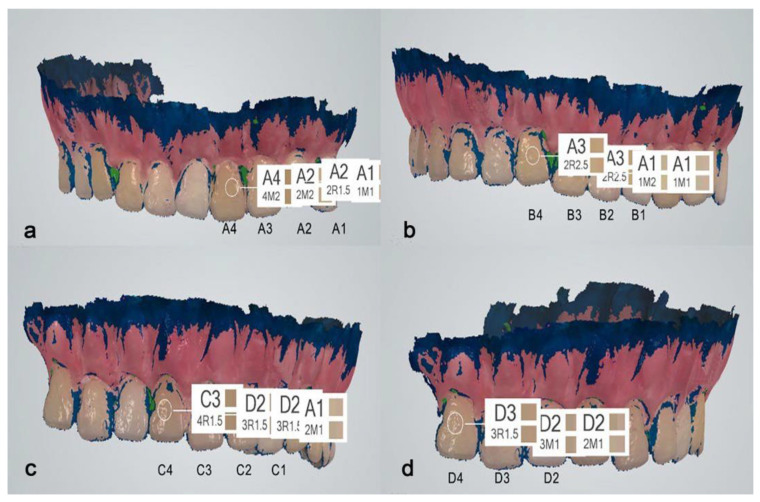
Shade selection of the middle third of the VC tabs using 3Shape TRIOS, according to VITA Classical Guide. (**a**) VC A group. (**b**) VC B group. (**c**) VC C group. (**d**) VC D group.

The color measurements of the study guide obtained with the CEREC Omnicam and 3Shape TRIOS are presented in Figure 9; Figure 10, respectively.

The color identification accuracy ranking is based on the number of shades identified by each intraoral scanner out of the 15 shade categories found in the VITA Classical color key, expressed as percentages. The benchmark represented by VITA Classical A (A1, A2, A3, A4), B (B1, B2, B3, B4), C (C1, C2, C3, C4), and D (D2, D3, D4) was chosen because it has the highest insertion, even at the present time, in color detection in everyday dental practice.

The results of shade detection using the VE, Omnicam, and TRIOS are illustrated in Table 1. Both primary and secondary results of the VE were considered. Out of the total of 15 shade tabs, the spectrophotometer managed to match only 3 shades (primary results) and 9 shades (secondary results). The Omnicam matched only three shades, whereas the TRIOS had four matches. The only shade that all devices matched correctly was A1. A2 tab was the only one to be matched three times, whereas tabs A3, B2, and B3 were matched twice. In contrast, tabs B1, C1, C2, and D4 were not correctly detected by any of the devices.

**Figure 11:**
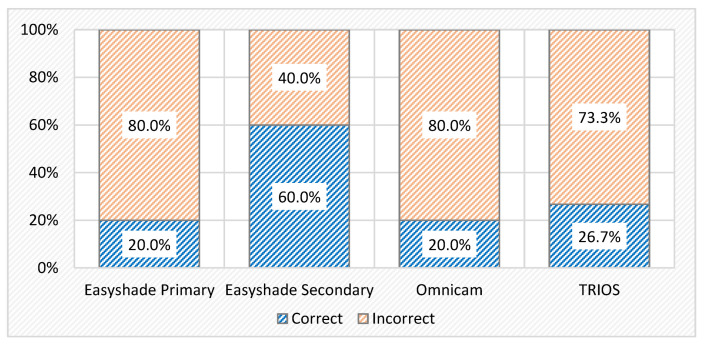
Comparison of accuracy between VE, Omnicam, and TRIOS.

The accuracy of the three devices when measuring the shade of the study model was calculated as a percentage (Figure 11). When comparing the primary results of the VE and the results obtained by the Omnicam and TRIOS, the latter is the most accurate (26.67%), whereas the other two scored 20%. When considering the secondary results of the VE, its accuracy (60%) is three times higher than that of the Omnicam. Statistical analysis showed no significant difference between the accuracy recorded by each of the four methods (Pearson chi-squared test, *p* = 0.054). There are, instead, statistically significant differences only between the VE—VE2 (*p* = 0.025*) and Omnicam—VE2 (*p* = 0.025*); the other methods compared two-by-two do not reveal statistically significant differences.

The L*a*b were individually compared for all four methods: VE, Omnicam, TRIOS, and Photoshop (Table 2).

Regarding the L* value, values recorded from Omnicam screenshots were the brightest, whereas TRIOS values were the lowest. The values obtained from the study model digital pictures were the most constant in terms of lightness values—2.066 dispersion—whereas the highest dispersion was recorded by the Easyshade spectrophotometer—6.444. There was a significant difference between the values recorded by the VE and the other three methods (*p* < 0.001). The post hoc Tukey HSD test shows that the VE had significantly different values than the Omnicam and TRIOS, while the comparison between values obtained by the Omnicam, TRIOS, and Photoshop also showed statistically significant differences.

As for the a* value, the Omnicam, TRIOS, and Photoshop methods recorded only positive values, except Easyshade, which also recorded negative values. This suggests that all recordings from the Omnicam, TRIOS, and Photoshop are located on the red side of the a* coordinate. The Omnicam recorded the highest values—10.27 on average—suggesting redder shades were detected. In contrast, the spectrophotometer obtained the closest values to the middle of the red-green color axis, with a dispersion of 1.417. However, the lowest dispersion was assigned to the TRIOS—1.318. The results showed again significant differences between the recordings with the VE and the other methods (*p* < 0.001). The post hoc Tukey HSD test shows that the VE has significantly smaller values than any of the methods. Moreover, it shows that there is also a significant difference between the values obtained by the Omnicam and TRIOS, as well as Photoshop. There is no significant difference between the TRIOS and Photoshop.

The b* value was investigated in a similar manner as the other two coordinates. The values recorded from Omnicam screenshots were the highest, 34.87 on average, whereas the average of TRIOS values was more than twice smaller. On the other hand, the TRIOS obtained the most constant values (the smallest spread), whereas the Omnicam recorded the highest variation, which was almost three times higher than that of the TRIOS. There was a significant difference between VE results and the other methods (*p* < 0.001). However, the post hoc test showed significant differences between the VE and Omnicam as well as the TRIOS. The values recorded by the Omnicam are also statistically significantly different in comparison with both the TRIOS and Photoshop methods, as well as in the case of the comparison between the TRIOS and Photoshop.

Furthermore, for each of the L*a*b values, the Pearson correlation coefficient was calculated in order to see whether there was any correlation between the results of the VE and the other methods for each of the three values (Table 3).

Regarding the L* value, the VE recorded the highest correlation with the Omnicam (r = 0.688), followed by the TRIOS (r = 0.595) and Photoshop (r = 0.444). All three correlations are positive; the first two are strong, the last one being moderate. As for the a* value, the VE recorded the highest correlation also with the Omnicam (r = 0.728), followed by the TRIOS (r = 0.716) and Photoshop (r = 0.566). All three correlations are strong and positive. Lastly, for the b* value, the VE recorded the highest correlation with the TRIOS (r = 0.898), followed by Photoshop (r = 0.874) and the Omnicam (r = 0.572). All three correlations are, again, strong and positive.

## 4. Discussion

The results found in this study support the rejection of the proposed null hypothesis because significant differences were highlighted regarding the accuracy of detecting dental shades among the VE and CEREC Omnicam and 3Shape TRIOS. Moreover, there was a significant difference between the L*a*b values obtained with the VE compared to the rest of the tested methods, but with one exception. Regarding the b* value, there was no significant difference between the VE and TRIOS results. The capacity of a spectrophotometer to detect the dental color is not more accurate than either of the two IOSs, the CEREC Omnicam and 3Shape TRIOS. Moreover, the individual performance of each device in determining the shades of the study model can be considered poor. All three of them scored less than 30% in matching the shades correctly. Surprisingly, the TRIOS had a better result than the spectrophotometer. On the other hand, despite the fact that the VE detected only 20% of the shade tabs correctly, its feature of suggesting the closest VITA Classical A1–D4 shade to the measure value appeared to be more efficient than the actual determination. This option was taken into consideration in the accuracy assessment analysis, but it did not generate a significant difference in comparison to the other devices.

As for CIELAB coordinates, considering the fact that other studies showed that the dental spectrophotometer is the most accurate device for teeth shade selection, VE L*a*b was compared to the other three methods [23,24]. Except for one out of nine comparisons, neither of the IOSs nor the Photoshop methods returned similar numerical values as the spectrophotometer. However, the correlation statistical test showed a strong, positive correlation between the values recorded by the VE and the other devices. This means that, even though the L*a*b values were different, the trend given by each method measuring each group of shade tabs was related to the CIELAB coordinates identified by the spectrophotometer. The color difference ΔE* was also calculated. All the values were higher than a previously proven level of clinically unacceptable mismatch, which is more than 2.72 ΔE* units for an in vitro study [25,26]. The closest values to the acceptable difference (ΔE*) were obtained through the Photoshop method, while the Omnicam method returned the highest color mismatches. For this reason, clinicians should not rely solely on an IOS accompanied by a picture analysis software for dental shade determination in the practice. The L* values showed that the Omnicam provided brighter measurements than the VE, while the TRIOS recorded darker values than the spectrophotometer. The VE was the only device that recorded negative a* values, whereas the Omnicam obtained more positive values, which can be translated into a more reddish hue. The TRIOS also recorded only positive a* values, but they had the least variation in comparison to the other methods. Nevertheless, the Omnicam returned the more positive b* values compared to the VE. All four methods scored positive values, while TRIOS b* values had the lesser yellowish hue. The significant difference between the results provided by the VE compared to the other methods were the high color differences ΔE*; however, correlated L*a*b values indicate that the actual methods influenced the measurements. A potential factor is the different ways in which the two programs, the VE itself and Photoshop, were able to interpret the images.

VITA Classical is the oldest commercial shade guide available on the market, and it is still highly used by dental professionals [27]. Due to the fact that both the spectrophotometer and IOSs are using VC as a reference guide, this conventional shade guide was used to test the accuracy of the devices. It is well known that VC has some disadvantages, such as illogical color distribution, and that it does not consider all Munsell color system characteristics, hue, value, and chroma [20,28]. However, these downsides were expected not to interfere with the results of the study.

Probably, the limitations of the guide influenced the outcomes. Even though all three devices are capable of detecting both natural and artificial teeth, the success rate of correctly matching the color of the tabs is surprisingly low. Whereas the spectrophotometer is considered the golden standard in dentistry, it scored only three out of fifteen possible matches, according to Table 1. Similar results were obtained by the two IOSs. Interesting was also the fact that some of the tabs could not be correctly interpreted by any of the devices. The current study did not assess the repeatability of the method, which probably would have shown whether the final results would be similar after repeated measures. Further investigations would be recommended in order to find out why there was such poor performance from the devices that have been tested and proven in previous studies. The procedure of shade detection was performed according to the manufacturer’s instructions for all three devices. There was a lack of quality in the build of the shade guide materials, or its design was not totally related to the aspect of natural teeth, or the devices were not able to detect the guide they were actually using as a reference. The difficulty with which the spectrophotometer and IOSs identified the correct color of the shade tabs shows that VC is potentially not a reliable shade guide, and it could explain why clinicians are also facing similar problems when using the guide for aesthetic purposes [29].

The accuracy of shade recording depends on a combination of factors in which elements dependent on human nature play a particularly important role, while at the same time having a number of subjective valences. We can mention a number of operator-dependent elements, among which experience can play an important role, without overlooking the time of day on which the recording is made.

However, this part could also be further examined from the repeatability point of view because the procedure was performed by one person, and errors could easily occur. One of the potential errors in the current study regarding color determination could be that the study model was manipulated by hand when examining it with the spectrophotometer, and that could influence the precise positioning of the device on the tooth. Moreover, the color was analyzed from the middle third of each tooth, but there was no certainty because a unique area was used for all the devices. These errors could be diminished by doing repeated measures.

Given the fact that all three devices used in the present study are also able to refer to the VITA Toothguide 3D-Master guide, the same study could be conducted, but replacing VC with TG. Reproducing the study with a different shade guide would show whether the outcomes are any different. Another possibility to test the used method would be through an in vivo study, examining natural teeth instead of artificial teeth, as the main purpose of the VE spectrophotometer is to determine the color of natural teeth.

The results obtained for the second method used in the study showed a significant difference in the L*a*b values recorded by the spectrophotometer and the other devices. None of the two IOSs were able at the time of the study to determine the color values according to CIELAB system by themselves. This would represent a limitation of the method. If, in the near future, this feature will be added to the software of scanners, then the study method can be reconsidered. In order to compensate for the unavailability of this software option, an alternative method was used. The results provided by the Photoshop method were significantly different than the ones recorded by the spectrophotometer. However, there was a strong, positive correlation between the results obtained through different methods on the same shade tabs. This means the shade tabs’ L*a*b coordinates were perceived in a similar manner by the different methods used, but there were other factors that influenced the outcome of each method. One of them has already been mentioned previously. Moreover, there could be a difference in the way of analyzing and estimating the CIELAB values. Whereas for the digital pictures of the study model, an 18% grey card was used for contrast, there was no possibility to include such a device in the scans. Even though the software of both IOSs uses a grey background, neither of them was as grey as the contrast card, and this might have influenced the digital analysis. Nevertheless, for this method, multiple measures would also be recommended.

The demand for better dental aesthetics is increasing and has prompted manufacturers to introduce better solutions to solve this challenge. Most studies have already demonstrated that instrumental methods are more accurate and objective in comparison to visual methods or studies that did not find any significant difference between these methods. As for IOSs and their capacity to detect dental shades, the current literature is still limited [30]. Therefore, the current study could be a relevant asset to this topic.

A study conducted in 2016 by Parameswaran et al. compared the accuracy of the VITA Easyshade spectrophotometer and visual methods. The VE scored only 25% accuracy in detecting shades from the VITA Classical guide [31]. The present study obtained only 20% accuracy for the same method. This would suggest that the spectrophotometer has limitations in assessing the color of artificial teeth, which was one of the objectives of this research. In contrast, another study that performed a similar method obtained 100% accuracy when measuring VC tabs with the VITA Easyshade [32]. However, the number of papers to contain information about this particular method is limited; therefore, no conclusion can be drawn.

A similar study also assessed the accuracy of several IOSs, including the TRIOS, Omnicam, and Easyshade V, but on VITA Mark II ceramic blocks [33,34,35]. The results indicated a significant difference between the VE and Omnicam but did not show any significant difference between the VE and TRIOS, similar to the findings of the current study. They were in agreement with previous literature, which also concluded that the TRIOS had comparable accuracy to the VITA Easyshade [36]. The individual accuracy values were higher overall: VE (78%), TRIOS (66%), and Omnicam (57%). In the present study, the TRIOS scored better (26.67%) than both the VE and Omnicam, which scored 20%. The different order and higher values of the other study might be because of the different materials used to test accuracy. Ebeid et al. used more aesthetic materials than the ceramic used in VC shade tabs [37].

The limitations of the study include, firstly, the use of the VC shade guide on a study model and the fact that it was an in vitro study. It is recommended to reconsider the study using, instead of VC, either TG or natural teeth. In this way, it could be demonstrated that the accuracy of the VE or IOS in shade detection is material dependent.

The color selection accuracy when using intraoral scanners can be influenced by a wide palette of external factors in which ambient light sources can play their extremely important role as well as elements of incorrect handling of the intraoral scanner. For future research directions on this extremely complex and growing topic, in accordance with the evolution of intraoral scanning types, we aim to corroborate the registration accuracy with the different times of the day, the ambient light with the degree of experience in the field of the one performing the registration, and the setting to the 3D Vitamaster color guides.

The aim of this study was to assess the accuracy of IOSs in comparison with the VE. Results could be improved and become more relevant if multiple measurements were performed for each method used. Also, in further research, the repeatability of these methods could be considered.

Another limitation consists of the absence of a software feature that allows either the CEREC Omnicam or 3Shape TRIOS to determine the L*a*b values by themselves. In order to compensate for this shortcoming, Adobe Photoshop was used to analyze screenshots of the scan models and digital pictures. However, both types of digital pictures would require a calibration process that would allow a more objective comparison among themselves and also with the VE values.

## 5. Conclusions

Within the limitations of this study, the aim and the objectives were reached. No significant difference was established between the accuracy of the VE and the two IOSs in determining the shade of the study model. Moreover, there was no significant difference between the two IOSs either. However, the poor rate of successfully matching the colors of the tabs exposed the potential limitations of either the VC itself or the VE and IOSs on detecting the shade of artificial teeth.

The first objective was accomplished, but the results were different than the expectations. The VE returned only 20% accuracy in detecting the VC color shade tabs. However, due to its software, it was able to suggest the closest VC A1–D4 related to the initial color measurement. Considering this feature too, the accuracy increased to 60%. Nevertheless, these values are still unexpectedly low for a device that is thought to be the golden standard in dentistry for detecting the shade.

The next objective was also reached. As for the VE’s results, the accuracies described by the Omnicam and TRIOS did not meet the expectations. The Omnicam scored only 20%, while the TRIOS scored 26.67%. Despite the fact that the TRIOS appeared to be a little more accurate, there was no significant difference between the two IOSs.

A significant difference between CIELAB values was also recorded between the VE, Omnicam, TRIOS, and the fourth method, Photoshop. However, the statistical test showed a strong, positive correlation for each of the three values between the VE and any of the methods. Moreover, the color difference ΔE*, regardless of the comparison group, was higher than 2.72 ΔE* units, which represents the acceptable upper limit for mismatch for an in vitro study.

The study also revealed the limitations of the instrumental devices that were used. First, both the VE and IOSs obtained unexpectedly low accuracy results. Possibly, the material VC is made of influenced the final accuracy values, but in practice, on a daily basis, dental materials represent a factor that cannot really be controlled. The IOSs are missing the CIELAB system from their software for now, but if it becomes available, it will certainly improve the shade detection step in aesthetic dentistry.

## Figures and Tables

**Table 1:** VITA Classical values of the SM measured with VITA Easyshade V, Omnicam, and TRIOS; SM—study model; VE—Easyshade primary results; VE2—Easyshade secondary results; OM—Omnicam results; TR—TRIOS results (the matches are highlighted in red, the mismatches are highlighted in blue).

SM	A1	A2	A3	A4	B1	B2	B3	B4	C1	C2	C3	C4	D2	D3	D4
VE	A1	A2	A3	B4	A1	A2	A3	A3.5	B2	A3	C3	D4	A1	C4	B3
VE2	A1	A2	A3	B4	A1	B2	B3	B4	B2	D3	C3	C4	C1	D3	B3
OM	A1	B2	A2	A3.5	A1	B2	B3	B3	B1	A2	A3	D4	B1	A2	A3
TR	A1	A2	A2	A4	A1	A1	A3	A3	A1	D2	D2	C3	D2	D2	D3

**Table 2:** Summary of ANOVA tests for L*a*b values recorded by Easyshade, Omnicam, TRIOS, and Photoshop.

		ANOVA Test		Post Hoc Tukey HSD	
	N	m ± SD	*p*-Value		*p*-Value
L—values				Easyshade–Omnicam	<0.001 **
Easyshade	15	75.67 ± 6.444	<0.001 **	Easyshade–TRIOS	<0.001 **
Omnicam	15	83.40 ± 2.772		Easyshade–Photoshop	0.931
TRIOS	15	68.93 ± 2.939		Omnicam–TRIOS	<0.001 **
Photoshop	15	76.53 ± 2.066		Omnicam–Photoshop	<0.001 **
				TRIOS–Photoshop	<0.001 **
a—values				Easyshade–Omnicam	<0.001 **
Easyshade	15	1.45 ± 1.417	<0.001 **	Easyshade–TRIOS	<0.001 **
Omnicam	15	10.27 ± 3.173		Easyshade–Photoshop	<0.001 **
TRIOS	15	8.17 ± 1.318		Omnicam–TRIOS	0.033 *
Photoshop	15	6.73 ± 1.710		Omnicam–Photoshop	<0.001 **
				TRIOS–Photoshop	0.232
b—values				Easyshade–Omnicam	<0.001 **
Easyshade	15	20.37 ± 4.604	<0.001 **	Easyshade–TRIOS	0.044 *
Omnicam	15	34.87 ± 6.760		Easyshade–Photoshop	0.585
TRIOS	15	15.83 ± 2.901		Omnicam–TRIOS	<0.001 **
Photoshop	15	22.50 ± 3.041		Omnicam–Photoshop	<0.001 **
				TRIOS–Photoshop	0.001 **

* *p* < 0.05 statistically significant; ** *p* < 0.01 statistically highly significant.

**Table 3:** Correlation coefficients between L*a*b values recorded by Easyshade, Omnicam, TRIOS, and Photoshop.

	Pearson’s r Correlation Coefficient	95% CI for r	*p*-Value
L*VE vs. L*Omnicam	0.688	0.271 ÷ 0.887	0.005 **
L*VE vs. L*TRIOS	0.595	0.118 ÷ 0.848	0.019 *
L*VE vs. L*Photoshop	0.444	−0.089 ÷ 0.779	0.098
a*VE vs. a*Omnicam	0.728	0.344 ÷ 0.903	0.002 **
a*VE vs. a*TRIOS	0.716	0.323 ÷ 0.899	0.003 **
a*VE vs. a*Photoshop	0.566	0.075 ÷ 0.836	0.028 *
b*VE vs. b*Omnicam	0.572	0.085 ÷ 0.839	0.026 *
b*VE vs. b*TRIOS	0.898	0.714 ÷ 0.966	<0.001 **
b*VE vs. b*Photoshop	0.874	0.654 ÷ 0.957	<0.001 **

* *p* < 0.05 statistically significant; ** *p* < 0.01 statistically highly significant.

## Data Availability

The data presented in this study are available on request from the corresponding author. The data are not publicly available due to ethical and privacy restrictions.

## Abbreviations

The following abbreviations are used in this manuscript:

3D	Three-dimensional
IOS	Intraoral scanner
OM	Cerec Omnicam
PNG	Portable network graphics
SM	Study model
TG	Toothguide VITApan 3D-Master
TR	3Shape TRIOS
VC	VITA Classical
VE	VITA Easyshade V
VE2	VITA Easyshade—secondary results
